# Systems View of Deconditioning During Spaceflight Simulation in the PlanHab Project: The Departure of Urine ^1^ H-NMR Metabolomes From Healthy State in Young Males Subjected to Bedrest Inactivity and Hypoxia

**DOI:** 10.3389/fphys.2020.532271

**Published:** 2020-12-07

**Authors:** Robert Šket, Leon Deutsch, Zala Prevoršek, Igor B. Mekjavić, Janez Plavec, Joern Rittweger, Tadej Debevec, Ola Eiken, Blaz Stres

**Affiliations:** ^1^Department of Animal Science, Biotechnical Faculty, University of Ljubljana, Ljubljana, Slovenia; ^2^Department of Automation, Biocybernetics and Robotics, Jožef Stefan Institute, Ljubljana, Slovenia; ^3^National Institute of Chemistry, NMR Center, Ljubljana, Slovenia; ^4^German Aerospace Center, Institute of Aerospace Medicine, Muscle and Bone Metabolism, Köln, Germany; ^5^Faculty of Sports, University of Ljubljana, Ljubljana, Slovenia; ^6^Department of Environmental Physiology, Swedish Aerospace Physiology Centre, KTH Royal Institute of Technology, Stockholm, Sweden; ^7^Faculty of Civil and Geodetic Engineering, Institute of Sanitary Engineering, University of Ljubljana, Ljubljana, Slovenia; ^8^Laboratory for Clinical Toxicology, Faculty of Medicine, University of Ljubljana, Ljubljana, Slovenia; ^9^Department of Microbiology, University of Innsbruck, Innsbruck, Austria

**Keywords:** urine, metabolome, NMR, inactivity, interplanetary travel, medicine, deconditioning, inflammation

## Abstract

We explored the metabolic makeup of urine in prescreened healthy male participants within the PlanHab experiment. The run-in (5 day) and the following three 21-day interventions [normoxic bedrest (NBR), hypoxic bedrest (HBR), and hypoxic ambulation (HAmb)] were executed in a crossover manner within a controlled laboratory setup (medical oversight, fluid and dietary intakes, microbial bioburden, circadian rhythm, and oxygen level). The inspired O_2_ (F_i_O_2_) fraction next to inspired O_2_ (P_i_O_2_) partial pressure were 0.209 and 133.1 ± 0.3 mmHg for the NBR variant in contrast to 0.141 ± 0.004 and 90.0 ± 0.4 mmHg (approx. 4,000 m of simulated altitude) for HBR and HAmb interventions, respectively. ^1^H-NMR metabolomes were processed using standard quantitative approaches. A consensus of ensemble of multivariate analyses showed that the metabolic makeup at the start of the experiment and at HAmb endpoint differed significantly from the NBR and HBR endpoints. Inactivity alone or combined with hypoxia resulted in a significant reduction of metabolic diversity and increasing number of affected metabolic pathways. Sliding window analysis (3 + 1) unraveled that metabolic changes in the NBR lagged behind those observed in the HBR. These results show that the negative effects of cessation of activity on systemic metabolism are further aggravated by additional hypoxia. The PlanHab HAmb variant that enabled ambulation, maintained vertical posture, and controlled but limited activity levels apparently prevented the development of negative physiological symptoms such as insulin resistance, low-level systemic inflammation, constipation, and depression. This indicates that exercise apparently prevented the negative spiral between the host’s metabolism, intestinal environment, microbiome physiology, and proinflammatory immune activities in the host.

## Introduction

Metabolomics has developed into a technology-driven discipline enabling improved data collection, analysis, and interpretation. In particular, ^1^H-NMR spectroscopy has received significant attention since it is non-destructive, non-biased, quantitative, and at the same time requires no sample derivatization (Emwas et al., 2019), is reproducible, quantitative, and enables identification of unknown novel compounds routinely in complex biological systems, such as human body or built environments (Murovec et al., 2018; Sket et al., 2018; Emwas et al., 2019).

The PlanHab project encompasses the two faceted nature of spaceflight, where human physiological responses are coupled to microbial responses to inactivity on one side and 21-day (prolonged) confination within built environment, similar to hospital settings, on the other (Debevec et al., 2014a; Simpson et al., 2016). The combined effects of 21-day inactivity/unloading and hypoxia were investigated in a controlled manner (crossover design) using medically prescreened cohort of healthy male volunteers. The experiment was executed adopting the European Space Agency (ESA) and NASA core bedrest data collection SOP (Standardization of bedrest study conditions 1.5, August 2009) controlling a number of parameters such as atmospheric oxygen content, levels of exercise (immobilization), daily water and nutritional intake, circadian rhythm, and microbial ambiental and aerosol bioburden next to the 24/7 medical surveillance (Debevec et al., 2014a; Simpson et al., 2016). In this study, the PlanHab repertoire of exploration was extended by analyses of urine ^1^H-NMR metabolomes during the run-in (5 day) and three consecutive experimental phases [21-day normoxic bedrest (NBR), hypoxic bedrest (HBR), and hypoxic ambulation (HAmb)] in healthy male test participants. Bedrest approach in experiments is widely adopted to simulate the effects of microgravity on various physiological systems of human body, especially for studies of bone, muscle, and the cardiovascular system by NASA, ESA, and Roscosmos (Hargens and Vico, 2016; Sundblad et al., 2016). On the other hand, physical inactivity in general has emerged as the fourth leading behavioral risk factor for worldwide mortality (Kelly et al., 2020). Risk of over 20 chronic conditions [e.g., coronary heart disease, stroke, type 2 diabetes, some cancers, obesity, mental health problems (e.g., depression), and neurological conditions (e.g., dementia)] is increased by physical inactivity making lack of exercise a global health problem (Kelly et al., 2020).

The past findings obtained within the PlanHab platform showed that a number of negative physiological symptoms related to obesity and metabolic syndrome developed in a dose-dependent manner over the course of 21-day experimental period in the HBR and NBR but were absent from the HAmb variant (Debevec et al., 2014b, 2016b; Rittweger et al., 2016; Simpson et al., 2016; Stavrou et al., 2016; Sket et al., 2017a, b; Strewe et al., 2017). In addition, the observed negative physiological symptoms faded effectively in 14, 10, and <4 days for HBR, NBR, and HAmb, respectively (Debevec et al., 2014b; Sket et al., 2017a, b). Also, many of the microbial parameters such as butyrate producing microbial community, the general bacterial and archaeal microbial communities were shown to respond to modifications in human intestinal environment but lagged behind the changes in human physiology and intestinal environment (Sket et al., 2017a, b). These findings suggested a time-dependent and complex interplay between the host physiology (including apparent constipation), immunity (inflammation), controlled diet, intestinal environment variables, and microbiome physiology in absence of exercise. The analyses of microbiome and associated environmental parameters suggested that the onset of inactivity gave rise to progressive shifts in intestinal environment boiling down to modified microbial metabolic activity and increased metabolism toward degradation of host mucus layer in bedrest variants (HBR, NBR) (Sket et al., 2017b). On the other hand, in the absence of such changes the healthy HAmb variant was coupled to the production of beneficial indole derivatives (Sket et al., 2017b). Further metagenomic analyses within the PlanHab platform (Sket et al., 2018) confirmed that inactivity and hypoxia resulted in a significant increase in the relative abundance of genus *Bacteroides* in HBR next to *Bacteroides* cell wall, capsule, virulence, defense, and mucin degradation genes [beta-galactosidase (EC3.2.1.23), α-L-fucosidase (EC3.2.1.51), Sialidase (EC3.2.1.18), and α-*N*-acetylglucosaminidase (EC3.2.1.50)] and genes coding for iron acquisition and metabolism proteins (Sket et al., 2018). In contrast, the corresponding microbial fecal metabolomes, intestinal chemical and metal profiles, and the diversity of bacterial, archaeal, and fungal microbial communities were not significantly affected within the timeframe using the experimental set-up of the PlanHab project (Sket et al., 2018). The fact that the genus *Bacteroides* and proteins involved in iron acquisition and metabolism, cell wall, capsule, virulence, and mucin degradation were also enriched at the end of HBR revealed that significantly increased constipation and electrical conductivity led to decreased intestinal metal availability that consequently affected the expression of codependent and coregulated genes in *Bacteroides* genomes. Data integration utilizing Bayesian network analysis resulted in the establishment of the first hierarchical model describing the onset of inactivity-mediated deconditioning over time (Sket et al., 2018).

The PlanHab wash-out period corresponded to a reintroduction of exercise, vertical position, and posture maintenance that resulted in stepwise amelioration of the negative physiological symptoms, indicating that physical activity as such introduced changes into the crosstalk between the host physiology, microbial physiology, mucin degradation, and proinflammatory immune activities within the host (Sket et al., 2017a, b, 2018). This observation was based on the fact that the observed progressive decrease in some of the parameters (e.g., defecation frequency, intestinal indole content) and concomitant increase in other (e.g., intestinal electrical conductivity, inflammatory markers) preceded or took place in absence of significant changes at the levels of microbial taxonomy, the corresponding functional genes, intestinal metabolomes, and accompanying metal profiles (Sket et al., 2017a, b, 2018).

Metabolic signal can be divided into three categories, human, microbial, and human-microbial cometabolites (Dumas et al., 2017; Wilmanski et al., 2019) and can represent a significant portion of dissolved organic matter in blood and urine. Hence, the selection of metabolomics layer for in-depth analysis of the PlanHab project-derived urine samples thus represents a logical continuation of efforts to discern and improve our understanding of the timing and the consequences of 21-day inactivity and hypoxia on human pathophysiology.

As there is a lack of data and understanding on the progressive changes in human metabolic responses coupled to microbial metabolites in the absence of exercise, we hypothesized that reduction in physical activity (complete inactivity) would (i) result in structured and significant changes in urine metabolomes of healthy participants; (ii) enable identification of significant groupings of experimental variants; (iii) provide discriminant metabolites between observed physiological states; (iv) enable the construction of metabolic network of co-occurring metabolites; (v) provide insight into the time-dependent changes in metabolomes; and finally (vi) enlighten the significantly different metabolic pathways between the experimental variants and also relative to the healthy initial state. In addition, the systemic hypoxia due to inactivity (HBR) versus ambulation in hypoxia (HAmb) was predicted to be an additional important factor aggravating the observed physiological changes within the 21-day PlanHab execution, unraveling the difference due to retained physical activity levels, hydrostatic pressures, and posture-related muscle activity in HAmb (Debevec et al., 2014a; Miles-Chan and Dulloo, 2017; Sket et al., 2017a, b).

## Methods

### Experimental Setup

Experimental setup, registration, approval, recruitment, medical prescreening, acquisition of clinical data and supervision, and hypoxic facility next to the detailed outline of the PlanHab study were prepared and conducted according to the European Space Agency’s standardization plan for bedrest studies (ESA, 2009), including sample size calculation and were extensively detailed before (Debevec et al., 2014a, 2016a; Rittweger et al., 2016; Simpson et al., 2016; Sket et al., 2017a, b, 2018; Stavrou et al., 2016; Strewe et al., 2017). In short, for this study, each healthy male, participant, characterized by numerous clinically relevant measurements to assert absence of disease with a state of physical, mental, and social welfare, underwent 5 days of baseline data collection during which participants were ambulant, 21 intervention days and 5–14 days of medical follow-up. The participants underwent the following three protocols: (1) normobaric NBR (fraction of inspired O_2_ (F_i_O_2_) = 0.209; partial pressure of inspired O_2_ (P_i_O_2_) = 133.1 ± 0.3 mmHg); (2) normobaric hypoxic ambulatory confinement (HAmb; F_i_O_2_ = 0.141 ± 0.004; P_i_O_2_ = 90.0 ± 0.4 mmHg; ∼4,000 m simulated altitude); and (3) normobaric HBR (F_i_O_2_ = 0.141 ± 0.004; P_i_O_2_ = 90.0 ± 0.4 mmHg; ∼4,000 m simulated altitude). Altogether, 11 healthy men underwent all three campaigns in randomized crossover design of PlanHab project. Subjects were enrolled by project manager and randomly allocated between campaigns using Latin square design method. Sample size was determined based on previous reports on bedrest studies to obtain sufficient predictive power ≥ 0.80 (Traon et al., 2007; Angerer et al., 2014; Debevec et al., 2014a, 2016a,b; Simpson et al., 2016; Sundblad et al., 2016). For detailed experimental protocols, please see Debevec et al. (2014a); Sket et al. (2017b). In essence, the combined effects of 21-day complete inactivity and hypoxia on healthy participants were examined within the PlanHab study utilizing 11 healthy medically prescreened participants in the crossover design under strictly controlled conditions according to ESA/NASA core bedrest data collection SOP in order to determine significant differences between samples and experimental variants relative to healthy baseline data collection.

### The PlanHab Project Acquisition of Clinical, Exercise, Dietary, and Ambiental Data

Acquisition of clinical, exercise, dietary, and ambiental data were described in detail before (Debevec et al., 2014a, 2016a; Simpson et al., 2016). The in-house database (Sket et al., 2017b) containing over 13,000 entries based on all measured variables in the PlanHab experiment (i.e., clinical, inflammation, immune, human physiology, and nutrition data next to the experimental design and characteristics of the participants) was checked for consistency and updated with recent publications related to the PlanHab project (Debevec et al., 2014a, 2016b, 2018; Keramidas et al., 2016; Louwies et al., 2016; Rittweger et al., 2016; Rullman et al., 2016, 2018; Simpson et al., 2016; Morrison et al., 2017; Strewe et al., 2017; Salvadego et al., 2018; Sarabon et al., 2018; Stavrou et al., 2018a, b; Ciuha et al., 2020). The in-house database was used to identify parameters that differed significantly between the experimental variants over the course of the experiment as described before (Sket et al., 2017b, 2018). This resulted in 48 parameters describing diet, intestinal metabolites, immune, and chemical parameters next to human physiology that were significantly different between NBR, HBR, and HAmb variants (*p* < 0.05; corrected for multiple comparisons). These served as the basis for the linking of observed body deconditioning to urine metabolites observed in this study.

### Participants

After initial prescreening according to NASA and ESA guidelines for bedrest studies, the data of 11 participants that finished all three interventions were included in our analysis with the following baseline characteristics (mean ± SD): age = 27 ± 6 years; body mass = 76.7 ± 11.8 kg; stature = 179 ± 3 cm; BMI = 23.7 ± 3.0 kg m^–2^; body fat = 21 ± 5%; maximal oxygen uptake = 44.3 ± 6.1 ml kg^–1^ min^–1^ (Debevec et al., 2014a; Sket et al., 2017a).

### Sample Collection

Urine samples were collected aseptically on a daily basis in the early morning during the 5 days of run-in period and 21 days of intervention periods. In total, 523 samples were obtained, aliquoted, and frozen at −20°C for further analyses.

### Urine Metabolome Analysis Using Proton Nuclear Magnetic Resonance

Urine samples (600 μl) were centrifuged at 10,000 × *g* for 30 min at 4°C to remove fine particles. Samples were filtered through 0.22 μm HPLC-compatible filters (Millipore, Germany), 400 μl aliquots were mixed with 200 μl ^1^H-NMR buffer as described before (Beckonert et al., 2007) and stored at −20°C until analysis. Before analysis, samples were thawed at room temperature and centrifuged at 12,000 × *g* for 5 min at 4°C; 550 μl of each sample was transferred into 5 mm NMR tube as described before (Murovec et al., 2018).

Proton nuclear magnetic resonance (^1^H-NMR) spectra were acquired on an Agilent Technologies DD2 600 MHz NMR spectrometer equipped with 5 mm HCN Cold probe. 2D experiments were measured on Agilent Technologies (Varian) VNMRS 800 MHz NMR spectrometer equipped with 5 mm HCN Cold probe. All experiments were measured at 25°C. ^1^H-NMR spectra of the samples were recorded with spectral width of 9.0 kHz, relaxation delay 2.0 s, 32 scans, and 32 K data points. Water signal was suppressed using double-pulsed field gradient spin-echo (DPFGSE) pulse sequence. Heteronuclear single quantum coherence spectrum (HSQC) for ^1^H- and ^13^C-dimensions (2D NMR) was acquired with spectral widths of 9.0 and 40 kHz for ^1^H- and ^13^C-dimensions, respectively, and 1,536 complex points for ^1^H-dimension, relaxation delay 1.5 s, 160 number of transients, and 128 time increments. Total correlated spectrum (TOCSY) was measured with ^1^H spectral widths of 7.0 kHz, 4,096 complex points, relaxation delay 1.5 s, 32 number of transients, and 144 time increments. The ^1^H and 2D spectra were apodized with an exponential function and a cosine-squared function, respectively, and zero filled before Fourier transform. NMR spectra were processed and analyzed using VNMRJ (Agilent/Varian) and Sparky (UCSF) software and MestReNova.

The resulting spectra were consequently analyzed in two complementary ways: (i) human expert chemometric untargeted metabolomics, including 2D spectra, and (ii) targeted quantitative metabolomics using Chenomx NMR Suite version 8.3 (Chenomx, Inc.) For the latter, all spectra were randomly ordered for spectral fitting using ChenomX profiler. Metabolites were thus identified with the support of Chenomx Compound Library extended by Human Metabolome Data Base (Wishart et al., 2009; Markley et al., 2017), giving access to chemical shift profiles of 674 compounds used in analyses. The number of database derived chemical shift profiles of metabolites used in analyses was further decreased by the procedures described below.

### Bioinformatic and Statistical Analysis of Urine Metabolomes

Two different approaches to asymmetric sparse matrix data analysis were adopted (Legendre and Legendre, 2012), as each compound concentration was (i) normalized by dividing the measured concentration into the total concentration of all metabolites in that sample and (ii) by Box-Cox or log2 transformation (Sket et al., 2018). The metabolites that were present in less than 5% of the samples (i.e., < the size of the smallest experimental group of samples in analysis) were excluded from further analysis.

The significance of difference in the metabolic characteristics of various groups of samples was tested using ANOSIM, NP-MANOVA, expressed as an overlap in non-metric multidimensional scaling (nm-MDS) trait space using Gower and Euclidean distance measures, and finally the dimensionality reduction selected through stress function and inspection of Shepard’s plots of correspondence between target and obtained ranks. To analyze the relationship between starting and endpoints of each variant, and also between the endpoints of particular variants, a number of established approaches were used: weighted UniFrac, uweighted UniFrac, analysis of molecular variance (AMOVA), HOMOVA, LEfSe, indicator species, and Metastats tests with 999 permutations were used as implemented in mothur (Schloss et al., 2009). Multiple-group comparisons were performed using Benjamini-Hochberg false discovery rate (FDR). Multiple test correction (Benjamini and Hochberg, 1995; Benjamini and Yekutieli, 2001), was used as described before (Sket et al., 2017a, b, 2018).

Associations between urine metabolites were calculated using non-linear Spearman correlation as implemented in mothur (Schloss et al., 2009), and significant interactions (*p* < 0.005) were used for further network analysis. Software Cytoscape (Shannon et al., 2003) was used to create interaction networks between the significantly different groups of metabolomes identified in the previous section, giving thus rise to two groups: (i) the beginning of the experiment and endpoint of HAmb on one side and (ii) the endpoints of experimental variants NBR and HBR at the other. Network characteristics were described using parameters, e.g., clustering coefficient, number of nodes and edges, and network density next to centrality measures such as betweenness and closeness (Shannon et al., 2003).

Furthermore, a complementary analysis using a completely distinct analytical approach utilizing a dedicated MetaboAnalyst tool (Xia et al., 2009) was adopted. The supervised classification using random forest method and pathway mapping were utilized where measured metabolites were compared with human metabolome database for identification of the affected metabolic pathways (Wishart et al., 2007). Pathway enrichment analysis was performed using global ANCOVA and topology analysis using relative-betweeness centrality in MetaboAnalyst (Goeman et al., 2004; Chong et al., 2019).

The final type of analysis introduced a sliding window analysis of the relationships between the recorded metabolic profiles. Metabolomes belonging to a particular day over the run-in and experimental phase were binned together using window size of 3 days and the increment step size of 1 day. For each window, the urine metabolites and their distribution between samples were used to calculate the mean values of 3 days span for all three experimental variants (HBR, NBR, HAmb). Furthermore, the metabolic windows of 3 days calculated for different experimental variants were compared with the first 3 days of baseline data collection using permutational multivariate analysis of variance (PERMANOVA) tests with 9,999 permutations to assess the significance of differences between multiple-group comparisons and elucidate the possible trends in changes of significance within each and between different windows.

## Results

### The Extent of Body Deconditioning in the PlanHab Project

**FIGURE 1:**
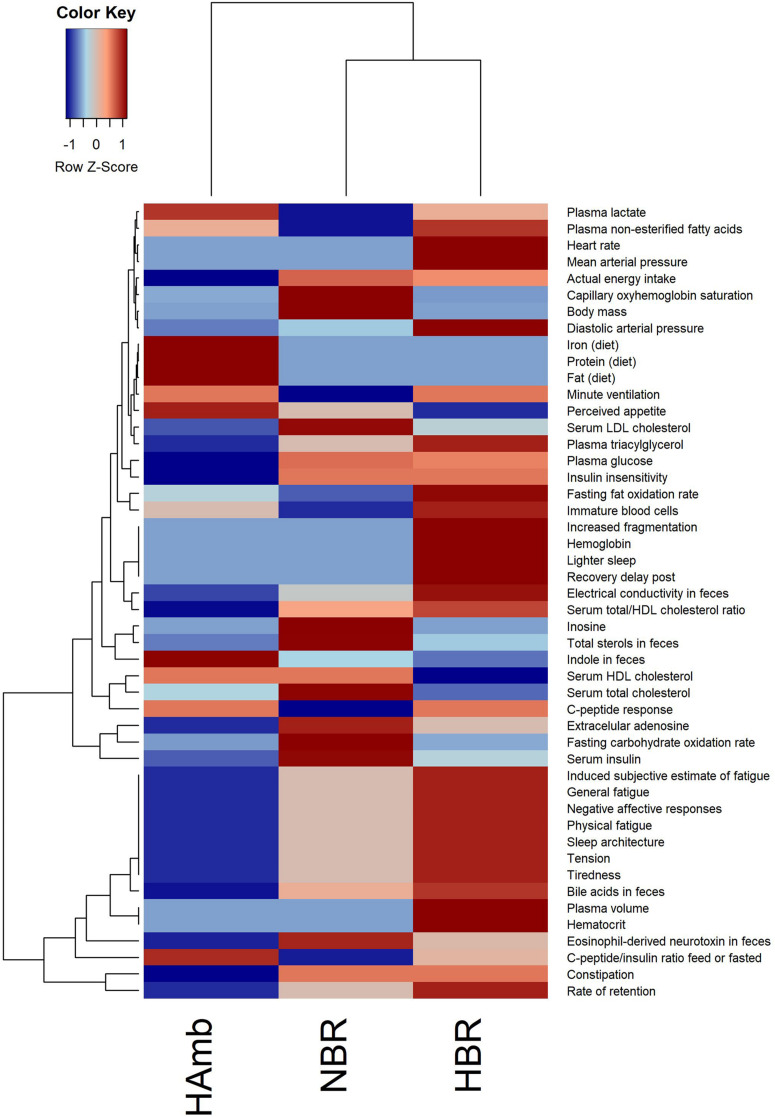
Heatmap plot showing the relationship between parameters describing human physiology, psychology, and intestinal environment that differed significantly at the end of the PlanHab experiment (*n* = 48; *p* < 0.05; FDR corrected) that are now part of the new version of the in-house PlanHab database (Sket et al., 2017b) based on all measured variables within the project. The inset to the left represents the magnitude of z-normalized data.

The in-house PlanHab database reported before (Sket et al., 2017b) enabled us to incorporate novel recently reported parameters within the PlanHab project (Supplementary Table 1) and identify 48 variables from other substudies within the PlanHab project that differed significantly between the experimental variants describing the clinical, inflammation, immune, human physiology, and nutrition characteristics of the participants (Figure 1). The results show clear separation between the HAmb variant and the inactive HBR and NBR variants. In addition, the variables were clearly separated into two broad response clusters with a number of variable subtypes, showing the complexity of the developed physiological and nutritional responses. The healthy levels of measured variables were retained for the major part of the measured variables in HAmb and hence constitute the least-affected phenotype, whereas those observed for HBR and NBR were classified as characteristic of insulin resistance (type 2 diabetes), low-level systemic inflammation, constipation, depression, symptoms related to metabolic syndrome, obesity, and body deconditioning due to inactivity. A number of specific changes can be observed in human physiology in response to either hypoxia or inactivity under hypoxia that are beyond the scope of this work and were already described in details within the PlanHab project publications (Sket et al., 2017b, 2018; Supplementary Table 1).

### Variations in Measured Urine Metabolites Between the Experimental Branches

**FIGURE 2:**
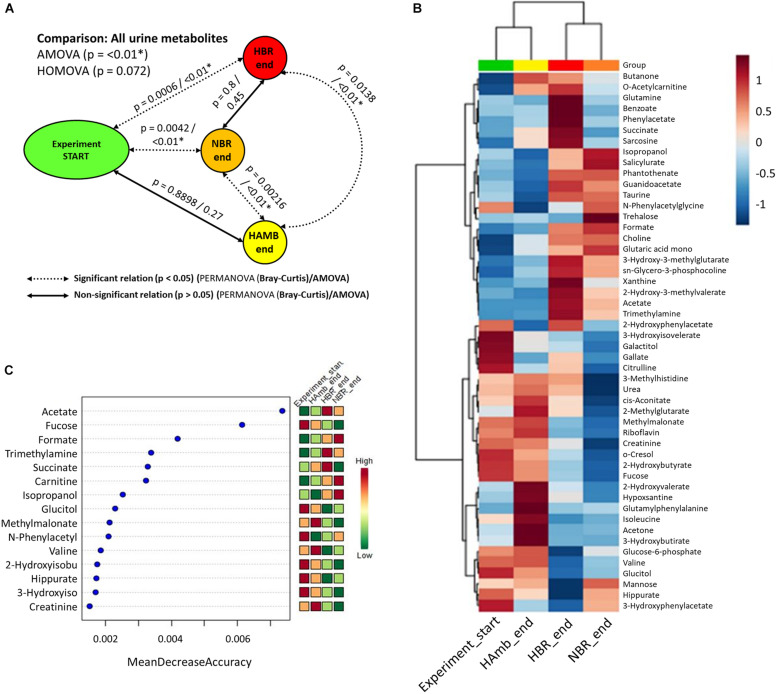
Schematic overview of the detected changes in urine metabolites. **(A)** Comparison of metabolite groups showing the start and HAmb, HBR, and NBR experimental variants using three different tests. The dotted and solid lines designate significant and non-significant differences between the groups. **(B)** Heatmap of the 50 most important urine metabolites according to AMOVA significance testing constructed using Euclidean distance measure and Ward clustering algorithm. **(C)** Graphical representation of 15 most informative metabolic features and their sample classification efficiency ranked by random forest algorithm. The insets to the right **(B,C)** represent the magnitude of z-normalized data.

Multiple comparisons using AMOVA test indicated significant shifts in metabolites between baseline data collection and endpoints of experimental variants (*p* < 0.01). Individually tested correlations between experimental variants showed that metabolites detected in HBR and NBR campaigns differed significantly from HAmb and baseline data collections (Figure 2A). As the metabolites detected in baseline data collection and HAmb group were not significantly different, these two groups represented rather healthy physiological signatures, as observed before in the PlanHab literature (Supplementary Table 1). Multiple comparisons of the most significant metabolites according to ANOVA significance testing (Figures 2B–C) confirmed the joint clustering of HAmb and baseline data collections as healthy physiological signatures on one side in contrast to HBR and NBR campaigns as affected states on the other. In this respect, the joint branching of the baseline data collection of healthy participants with HAmb variant represented thus the rather healthy human physiological signatures on one side with NBR and HBR experimental variants representing severely affected participants on the other (Figure 1; Supplementary Table 1).

The metabolites most involved in separation of the two experimental branches (healthy vs. affected) listed within Figures 2B,C represent the classes of microbial metabolites (e.g., acetate, formate, hippurate), human-microbe cometabolites (e.g., trimethyl amine, hippurate, carnitine, acetyl carnitine, cresol, phenyl acetyl glycine), and human-derived metabolites involved in ATP synthesis (e.g., creatinine, choline, guanidinoacetate, hypoxanthine, xanthine), DNA (purine) metabolism (e.g., uric acid, xanthine, hypoxanthine), tricarboxylic acid cycle (e.g., succinate, citrate), muscle mitochondria (e.g., isoleucine), generation of reactive oxygen species (ROS; e.g., xanthine, hypoxanthine), bile acid metabolism (e.g., taurine), and others. It can be seen that numerous metabolites were associated and could be hence involved with distinct complex physiological responses detailed in Figure 1.

Of interest, the three collections of run-in baseline data metabolomes obtained from healthy and medically prescreened participants were not significantly different (PERMANOVA test; *p* > 0.05; FDR corrected). This shows that urinary metabolomes obtained during the run-in baseline data collection were representative of healthy normal males.

### Interaction Network Analysis of Co-occurring Metabolites

**FIGURE 3:**
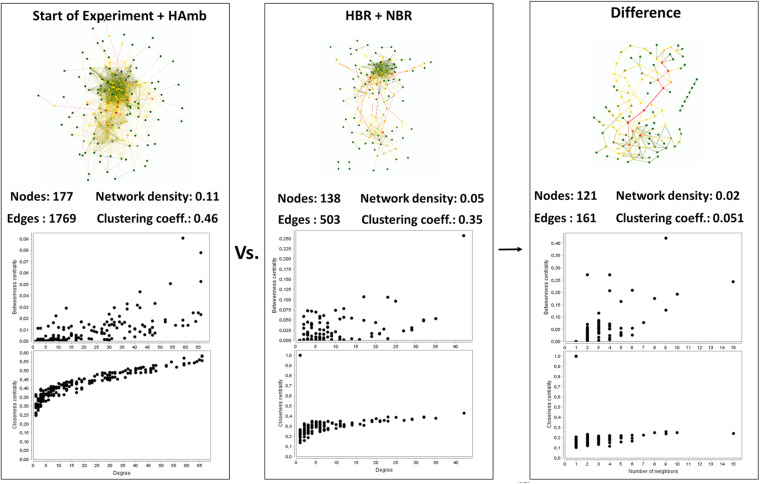
An overview of the complexity of the metabolic co-occurrence networks and their characteristics reported for the healthy (start; HAmb) and affected (NBR; HBR) metabolic states. The rightmost pane represents the difference between the two networks, showing the extent of lost metabolic interactions and nodes due to modifications in human physiology in response to conditions in HBR and NBR (inactivity and hypoxia).

Interaction network of metabolites characteristic of the healthy state showed us 177 statistically significant connected metabolites (i.e., nodes; Spearman correlation *p* < 0.005) with a total of 1,769 edges representing the co-occurrence patterns between metabolites (Figure 3). In contrast, the interaction network in affected participants of NBR and HBR variants showed a severe reduction of more than 30% in the number of statistically significantly connected metabolites and a 2.5 times reduced number of their interactions. This testifies that a reduction in physical exercise is coupled to significant reduction in metabolic diversity within human body.

Based on centrality measurements (betweenness, closeness), the most important metabolites representing the difference between healthy and affected states that were identified also using different statistical approaches (Supplementary Table 1) were enriched in either healthy or affected states (Figures 2B,C), suggesting significant shifts existed in the metabolic makeup of the human urine after introduction to inactivity within the PlanHab project and secondly very few to the project itself.

In addition, these graphical representations of metabolic co-occurrence networks clearly demonstrate the complexity of metabolic makeup of developed metabolic states observed in the PlanHab project showing that the search for a single or a handful of biomarkers would be prohibitive and oversimplification and that a more complex approach needs to be utilized to derive important information.

### Variations in Predicted Urine Metabolic Pathways

As many metabolites can be involved in different not necessarily complementary metabolic pathways, the collected metabolomics data were used to reconstruct the most important metabolic pathways contributing to the observed differences in metabolomes. The pathways were identified based on the importance of underlying metabolites (pathway impact) and the significance of comparison between different metabolites (significance after FDR).

**FIGURE 4:**
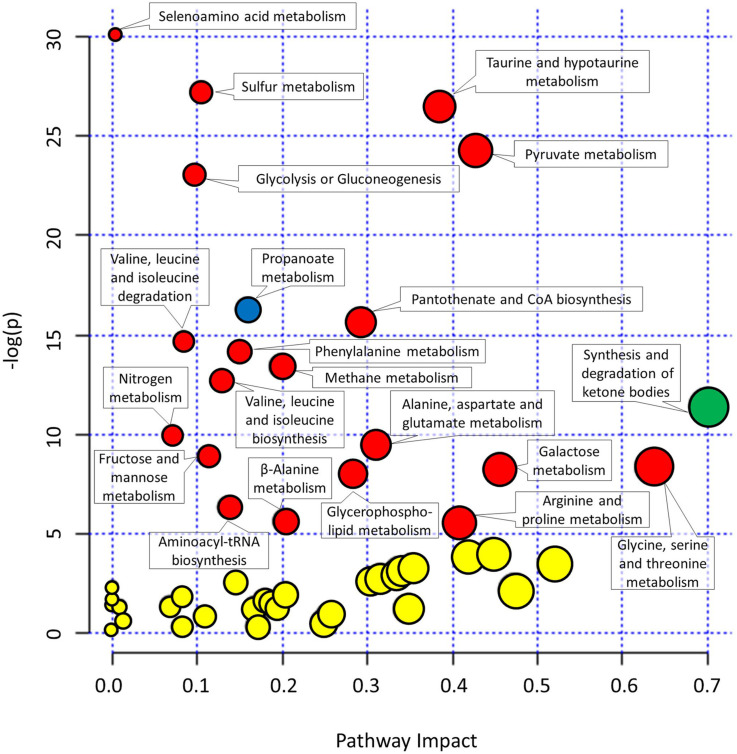
Graphical representation of metabolic pathways according to their significance of change between experimental variants relative to the start of the experiment [significance (−log(p))] and importance of metabolites within a given pathway. *y*-axis: the *p*-Values (−log(p)) from pathway enrichment analysis using Global test for testing differentially expressed metabolites. Significantly changed urine metabolic pathways were based on KEGG database relative to the start of the experiment. *x*-axis: pathway impacts from the topology analysis using relative-betweeness centrality were used to estimate the importance of measured metabolites within a given metabolic pathway. Designation of changes in metabolic pathways relative to the start of the experiment: yellow, not significant changes; red, significant changes in NBR and HBR; blue, significant changes in all three variants. See Supplementary Figure 1 and Supplementary Table 2 for additional information. The size of the circles corresponds to pathway impact (*x*-axis) for simplicity.

The metabolites involved in propanoate metabolism (*p* < 0.0001) were enriched in comparison with the start of the experiment (Figure 4 and Supplementary Table 2) in all three campaigns. On the other hand, the metabolites involved in synthesis and degradation of ketone bodies with pathway impact 0.7 were enriched solely in HAmb experimental variant (*p* < 0.0001) considering FDR but not in HBR and NBR variants.

Most enriched pathway in the most affected variant of the PlanHab project, the HBR campaign, were, e.g., glycolysis or gluconeogenesis and furthermore the concentration of glucose 1-phosphate were lower at the end of HBR in comparison with the start of the experiment, whether on the other hand metabolite acetate was increased in both HBR and NBR campaigns (Figures 3, 4). Acetate was the main factor in HBR and NBR campaigns suggesting the enriched pyruvate metabolism.

Other significantly affected metabolic pathways enriched in HBR and NBR in comparison with the start of experiment were aminoacyl-tRNA biosynthesis, arginine and proline metabolism, beta-alanine metabolism, fructose and mannose metabolism, galactose metabolism, glycerophospholipid metabolism, methane metabolism, nitrogen metabolism, pantothenate and CoA biosynthesis, selenoamino acid metabolism, sulfur metabolism, taurine and hypotaurine metabolism, valine, leucine, and isoleucine biosynthesis (Figure 4 and Supplementary Table 2).

Finally, an overview of the number of affected pathways suggested that the introduction of the participants into the PlanHab project significantly affected four metabolic pathways in HAmb in comparison with the starting metabolic makeup, whereas a five and eight times larger number of pathways were progressively affected in NBR (*n* = 22) and HBR (*n* = 32), respectively. This is in line with our observation that inactivity irrespective of hypoxia resulted in 30% reduction in the number of statistically significantly connected metabolites, a 2.5 times reduction in the number of interactions and that reduced physical exercise resulted in diminished metabolic diversity within human body.

### The Sliding Window Time-Frame Analysis

**FIGURE 5:**
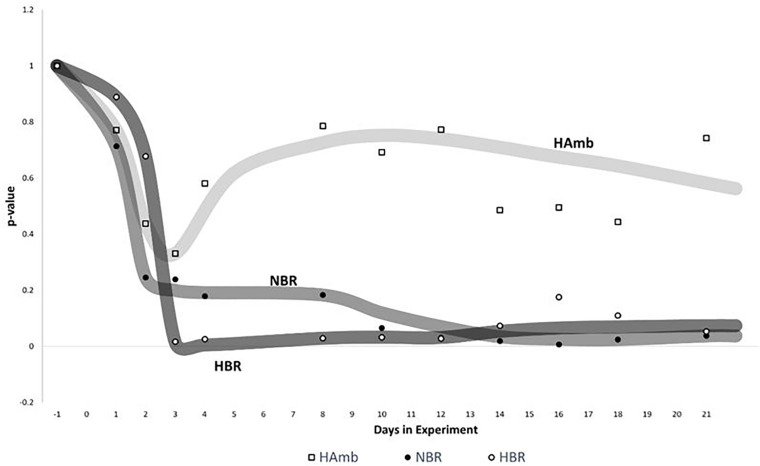
Analysis of significant changes in metabolic signatures over time. Sliding window analysis (*n* = 3) was adopted to elucidate the relationships between the recorded metabolic profiles. *x*-axis: time of metabolic signature. For each signature, metabolomes were binned together using window size of 3 days and the increment step size of plus 1 day. For each window, urine metabolites and their distribution between samples were used to calculate the mean values of 3 days span for all three experimental variants (HBR, NBR, HAmb) and compared with the first 3 days of baseline data collection. *y*-axis: the significance of differences between different metabolic windows over time (*p* < 0.05). Multivariate non-parametric test PERMANOVA with 9,999 permutations was used to assess the significance of differences between multiple-group comparisons and to elucidate the possible trends in changes of significance within each and also between different windows.

Sliding window analysis enabled us to compare each bin of 3 days to the start of the experiment in order to identify the onset of significant changes in experimental variants (Figure 5) over time. The changes in metabolic makeup in both bedrest variants (HBR, NBR) deviated progressively away from the initial status until significant changes were detected by the end of the second week of the experiments. Significant changes in human urine metabolome were observed by the end of the first week in HBR, whereas the apparent delay of significant changes in NBR in comparison with HBR lasted till the day 12, and the difference can be attributed to the lower levels of oxygen in HBR. It is interesting to note that the pattern of metabolome deviation of HAmb variant from its original state actually followed an acclimation pattern. The initial effects of hypoxia were thus ameliorated in HAmb by the retained levels of exercise in this particular variant of the PlanHab project, diurnal vertical posture maintenance activity, and hence establishment of hydrogradients within the HAmb, giving rise to overall insignificant changes in HAmb urine metabolites to the starting point during 21 days of the experiment.

## Discussion

The unique crossover design allowed us to include responses of the same participants to all three experimental variants, NBR, HBR, and HAmb, under the controlled nutritional, environmental, and experimental conditions. The same general trends of body deconditioning were recovered in this study based on ^1^H-NMR metabolomics of urine, as described before using different sets of markers and approaches in the PlanHab subprojects (Debevec et al., 2014a, 2016b; Rittweger et al., 2016; Simpson et al., 2016; Strewe et al., 2017, 2018; Stavrou et al., 2018a, b; Supplementary Table 1). This shows large congruence between the various independently collected datasets within the PlanHab and the metabolomics approach used in this study. For instance, zonulin concentration in blood samples (Strewe et al., 2018) showed the same patterns as zonulin samples collected from fecal samples (Sket et al., 2017b).

In this respect, our study demonstrates that ^1^H-NMR metabolomics coupled to standardized analytical approaches and sample preparation next to in-depth statistical analyses allows for comprehensive characterization of the physiological responses and enables the detection of subtle metabolic changes during the initial and reversible body deconditioning in response to 3-week inactivity. In comparison with HAmb, the participants involved in NBR and HBR exhibited specific and different metabolic trajectories giving rise to severely reduced metabolic diversity and hence the reduction in the number of metabolic pathways under controlled experimental and nutritional conditions (Figures 2, 3). In essence, this shows a profound impact of the onset of 3-week inactivity on human physiology revealing the progressive systemic maladjustments. Finally, the Bayesian modeling in our previous work (Sket et al., 2017a, b, 2018) showed that the significant changes in human physiology in the PlanHab project preceded or took place devoid of the corresponding changes at the level of intestinal microbiome. The genus *Bacteroides* and proteins involved in iron acquisition and metabolism, cell wall, capsule, virulence, and mucin degradation were enriched solely at the end of the third week in HBR only. Apparently, constipation and electrical conductivity decreased intestinal metal availability, induced modified expression of coregulated genes in *Bacteroides* genomes (Sket et al., 2018), possibly also the zwitterionic capsular polysaccharides with anti-inflammatory properties (Neff et al., 2016).

Our findings suggest that the decision of the host to minimize physical activity under hypoxic conditions (HBR) is detectable within a few days at the level of urine metabolites using ^1^H-NMR and by the end of the first 10 days in NBR irrespective of individual responses to food intake (Sato et al., 2018), daily composition, time of ingestion, and diurnal cycles of sleep described before (Sket et al., 2017a, b, 2018). Our results show reproducibly high flexibility of the underlying physiological metabolic pathways in the absence of the diurnal metabolic signals from the use of skeletal muscles (Schranner et al., 2020). This is important as in the absence of the metabolic signals from the use of human skeletal muscles, the metabolomes of other body organs seem to develop primarily different metabolic changes with little similar alterations that showcase the complexity of consequences due to the lack of exercise at the organismal level (Starnes et al., 2017).

This shows that the host’s metabolic and other physiological and psychological responses (Sket et al., 2017a, b, 2018; Figure 1) actually precede the responses of microbiome at the community structure level, but the chemical crosstalk between the two entities remains apparently responsive as based on the differences in metabolites that are known to be cometabolized by both and exchanged between the two subsystems (human and microbiome). Consequently, it is apparently the host that can be held responsible for the differences in thermodynamic niches provided to the microbes and to which microbial constituents respond. The colonic transit time was put forward as one of the most important parameters of intestinal tract related to bacterial metabolism and mucosal turnover in the gut (Roager et al., 2016), as also observed in our past studies (Sket et al., 2017a, b, 2018), and is hence a highly important factor to be considered in future metabolomics studies.

This complex crosstalk between microbiome and host’s systems is influenced by innumerable environmental parameters (Rooks and Garrett, 2016), crosstalk within microbial domains (Neff et al., 2016), and human evolutionary adaptations (Murray and Montgomery, 2014). In addition, their interaction can act locally and across greater distances within the human body, with some yet undetermined temporal delays (Rooks and Garrett, 2016). However, the contribution of microbiome to metabolic conversions of exercise-induced metabolites was shown to be of significant importance (Scheiman et al., 2019) acting as natural, microbiome-encoded enzymatic processes converting muscle lactate to formate and providing it back to host. In essence, this provides support for the concept, that mammals are holobionts, dependent on microbial and host genome information for optimal performance (Rooks and Garrett, 2016; Sket et al., 2018).

The approach adopted in this study provides an opportunity to generate new hypotheses on metabolic pathway perturbation. One can indeed hypothesize that the metabolites involved in metabolic pathways identified in this study in fact act as signaling molecules [or account for lack of these (e.g., in HBR, NBR)] involved in the PlanHab symptoms as detailed in Figure 1: insulin resistance, low-grade inflammation, different mitochondrial function, miRNA expression in large muscles, differences in lipid oxidation, mood changes, and depression (Debevec et al., 2014a, 2016b; Rittweger et al., 2016; Simpson et al., 2016; Sket et al., 2017a, b, 2018; Strewe et al., 2017, 2018; Stavrou et al., 2018a, b; Supplementary Table 1). In addition to those listed above, groups of metabolites identified in this study were also associated with: (i) the chronic obstructive pulmonary disease (COPD) (Adamko et al., 2015; Za̧bek et al., 2015) and included metabolites such as 3-hydroxyisovalerate, 2-hydroxyisobutyrate, creatinine, formate, taurine, urea, choline, isoleucine, pantothenate, valine, and its degradation to beta-aminoisobutyric acid during metabolism of branched-chain amino acids suggest increased catabolism associated with COPD; (ii) cardiovascular disease as a results of associated chain of events such as tissue hypoxia (gut ischemia) due to reduced oxidative phosphorylation and energy production that lead to pulmonary hypertension, systemic inflammatory responses, and increased risk of cardiovascular disease, type 2 diabetes, depression, and osteoporosis (Jones, 2014). Phospholipids such as trimethylamine (TMA), choline, and trimethylamine-*N*-oxide (TMAO) were strongly correlated with cardiovascular disease (Senn et al., 2012); and (iii) diabetes and the metabolic syndrome where different metabolites and metabolic pathways were correlated with the onset of the disease, such as isoleucine and phenylalanine, alanine, aspartate and glutamate metabolism, glycine serine and threonine metabolism, and phenylalanine metabolism (Wang et al., 2011; Jones, 2014).

In the single study of human metabolic responses to microgravity simulated in a 45-day 6° head-down tilt bedrest (HDBR) experiment (Chen et al., 2016) utilizing ^1^H-NMR in urine metabolomic analyses, similar changes in a limited number of biomarkers were detected (corresponding to NBR variant of our experiment), such as increased guanidinoacetate associated with enhancement of protein turnover inducing further muscle turnover, trimethylamines and taurine associated with cardiovascular diseases, and mammalian-microbial cometabolites such as acetate and hippurate, products of microbial fermentations, and dietary protein metabolism. This observation signifies congruent detection of a small number of the most informative metabolites in the two bedrest studies. However, it also shows that there is little congruency between different metabolomics studies based on the precise nature of a handful of specific metabolites to be assigned as specific biomarkers for certain disease or healthy status (Schranner et al., 2020). This is further exemplified by the incompatibilities between the methods, experimental designs, statistical approaches utilized (biomarker vs. pathways), levels of disease development, reversibility of the symptoms and conditions. However, the correspondence is markedly increased by the adoption of metabolite integration into metabolic pathways that are up- or downregulated, as shown in this study and in comparison to other studies utilizing the pathway approach where the same affected pathways have started to emerge for specific conditions (Sheedy et al., 2014; Elliott et al., 2015; Tynkkynen et al., 2019; Kelly et al., 2020).

From this it follows that no simple or single metabolic biomarker exists for delineation of particular human state (e.g., healthy vs. diseased in our experiment; trained vs. untrained; active vs. sedentary; young vs. old or any other group comparisons). In contrast, rather complex multivariate descriptions of metabolic makeup are needed to capture commonalities in human physiological states due to complex responses in human physiology, large interpersonal variability and variability over time, the fact that the same metabolites can act in different metabolic pathways and can hence act as up- or downregulated depending on the pathway.

Significant further research work will be needed to understand how the regulatory cascades of physical exercise and oxygen supply translate stimuli to various host’s tissues and microbiome domains that all affect human metabolic makeup and crosstalk between the domains of holobiont. The adoption of supervised and automated analyses amenable for re-analyses once improved algorithms, databases, statistical approaches arise enable us to continuously expand and learn from the datasets at hand over time. One has to realize that long-term bedrest studies with females are significantly more challenging and hence not many studies with sufficient statistical power were reported so far to close the gap. With the concomitant methodological development, the exploration of more complex female metabolome and responses to inactivity and hypoxia can be commenced, extending our recent FemHab work on this topic (Debevec et al., 2016a). Finally, genetic and environmental parameters likely play pivotal roles and further work is needed to understand their relative contributions, how these can be managed using metabolomics as one of the most promising approaches to explore these relationships (Kelly et al., 2020).

A few limitations and concepts of this study need to be considered. First, although the sample size utilized in this study seems relatively small from the perspective of screening random populations of participants, the sample size was well within the limits of recent detailed studies adopting the bedrest format or others (David et al., 2014a, b; Thaiss et al., 2014; Chen et al., 2016). Second, the effects of supposedly limited statistical power and accompanying potential for type-II error were at least partly alleviated by the fact that the test participant population was prescreened for healthy young males according to SOP used by ESA/NASA (Thevenot et al., 2015). Third, this study was conducted according to the European Space Agency’s standardization plan for bedrest studies (ESA, 2009), taking into account results of pre-experiments (Debevec et al., 2016b; Keramidas et al., 2016; Rittweger et al., 2016; Stavrou et al., 2016; Sket et al., 2017b; Strewe et al., 2017), Guidelines for Standardization of Bed Rest Studies in the Spaceflight Context (Angerer et al., 2014; Sundblad et al., 2016), and past recommendations on the sufficient sample size for measurements of the majority of routine parameters (Traon et al., 2007). Fourth, the PlanHab project was executed as crossover design experiment, hence the same participants were subjected to all experimental conditions in separate campaigns, further minimizing the overall interpersonal variability between campaigns.

In order to study metabolic deconditioning of the human body exposed to inactivity or other metabolic disorders, that may arise as a result of either acute or chronic and communicable or non-communicable diseases (Supplementary Table 1), the adoption of multivariate analysis of complex metabolomes in a unified framework can unravel more biologically relevant findings than search for a few biomarker metabolic or microbial species (Visconti et al., 2019; Kelly et al., 2020). In addition, ^1^H-NMR metabolomics offers quantitative insight (Beckonert et al., 2007; Emwas et al., 2019) as it is not compositional in contrast to shot-gun or amplicon metagenomics (unless deliberately transformed) (Vandeputte et al., 2017; Contijoch et al., 2019) and can be used in metabolic and computational modeling for guided decisions and health monitoring in personalized medicine approaches (Sung et al., 2016; Palumbo et al., 2018).

## Conclusion

The PlanHab project was designed to investigate in a controlled manner the combined effects of 21-day inactivity/unloading and hypoxia on a medically prescreened cohort of healthy male volunteers in crossover design. In total, 523 urine metabolomes were analyzed and processed using standard quantitative ^1^H-NMR approaches and ensemble of multivariate methods from three interventions: normoxic bedrest, hypoxic bedrest, and hypoxic ambulation. Results show that in contrast to hypoxic ambulation and run-in period inactivity alone or combined with hypoxia resulted in significantly reduced systemic metabolic diversity, increasing number of affected metabolic pathways, and faster metabolic deconditioning. The maintained vertical posture and controlled but limited activity in hypoxic ambulation variant prevented the development of negative physiological symptoms such as insulin resistance, low-level systemic inflammation, constipation, depression, symptoms of metabolic syndrome, and body deconditioning reported before in the PlanHab project. Metabolic and pathway diversity as a response to physical activity are apparently required to prevent the negative spiral between the host and microbiome physiology governed by intestinal environment and proinflammatory immune activities of the host. In order to study metabolic deconditioning of the human body exposed to inactivity or other metabolic disorders, the adoption of multivariate analysis of complex metabolomes in a unified framework of metabolic pathways can unravel more biologically relevant findings than a search for a few specific metabolic biomarker signatures.

## Data Availability Statement

The datasets generated for this study are available on request to the corresponding author.

## Ethics Statement

The studies involving human participants were reviewed and approved by REPUBLIC OF SLOVENIA Ministry of Health National Medical Ethics Committee Štefanova 5, 1000 Ljubljana, Slovenia, http://www.kme-nmec.si/kontakt/. The patients/participants provided their written informed consent to participate in this study.

## Author Contributions

BS provided the concept for metabolome analysis and drafted the manuscript. TD and JR collected the samples. BS, RŠ, and JP designed the metabolome analyses. RŠ, BS, ZP, LD, OE, and IM conducted the research. RŠ, BS, and LD analyzed the data. RŠ and BS provided necessary code to streamline ^1^H-NMR spectra analyses and provided statistical analyses. All authors provided intellectual content at various stages of project development and manuscript preparation and approved the final version of the manuscript.

## Conflict of Interest

The authors declare that the research was conducted in the absence of any commercial or financial relationships that could be construed as a potential conflict of interest.
